# American Academy of Dental Science

**Published:** 1853-04

**Authors:** 


*American Academy of Dental Science.*—A meeting of several dentists and physicians was held on the 7th of March, 1853, in Baltimore, for the purpose of taking into consideration the expediency of establishing an "American Academy of Dental Science." Prof. W. R. Handy was appointed Chairman, and Prof. P. H. Austen, Secretary. The meeting being thus organized, the advantages of such an organization, and best method of attaining the purposes contemplated by it, namely, the encouragement of improvements, discoveries and research in the science and art of dental surgery were briefly discussed. It being the opinion of the meeting that it might be made instrumental in the advancement of these objects, it was on motion, *Resolved*, that a memorial be addressed to the Maryland Legislature asking for a charter. Dr. T. E. Bond was chosen a committee to draft it and take the necessary steps for its procurement. The meeting now adjourned to meet on the 25th of March, at the Baltimore College of Dental Surgery.

Agreeably to adjournment, a number of gentlemen interested in the cause of dental science, assembled in the Chemical Hall of the College, at 11 o'clock, A. M., when, on motion, Dr. W. R. Handy was called to the chair, and Dr. H. Colburn, was appointed Secretary.

The meeting was addressed on the subject for which it had been called, by Drs. T. E. Bond, E. Townsend, W. H. Dwinelle, J. D. White, C. A. Harris, E. Parry, W. W. H. Thackston and R. Arthur.

On motion, it was *Resolved*, unanimously, that the present is a favorable time for establishing an Academy of Dental Science.

Dr. Austen made a verbal report of the proceedings of a committee appointed at the preceding meeting to draft the form of a constitution and by-laws.

The constitution drawn up by said committee, which consisted of Drs. Austen and Piggot, was read, and several of its sections amended and adopted.

The remaining sections, after some discussion, were referred to a committee consisting of Drs. Harris, Parry and Thackston, for revision, who were instructed to report at 4 o'clock, P. M. The meeting then adjourned until 4 o'clock, P. M.

The meeting assembled agreeably to adjournment, when the committee to whom was referred the sections of the constitution not considered at the morning session, presented a report, which was read, and on motion, accepted. But as many of the gentlemen composing the meeting were compelled to leave the next morning, it was thought there would not be sufficient time for the proper consideration of the report, it was, therefore, after some discussion,

***Resolved,*** That a committee of five be appointed to prepare a constitution and by-laws for the American Academy of Dental Science, to be presented at a meeting to be held in Baltimore on the first Tuesday in March, 1854; also, to issue a circular letter of invitation to such members of the profession as they may deem proper.

On motion, the Chair was authorized to appoint said committee, and, announced as such, Drs. C. A. Harris, R. Arthur, E. Parry, E. Maynard and W. W. H. Thackston.

The meeting now adjourned to the first Tuesday in March, 1854.

The meeting was attended by about thirty dentists and some four or five physicians.

